# Machine learning-assisted ammonium detection using zinc oxide/multi-walled carbon nanotube composite based impedance sensors

**DOI:** 10.1038/s41598-021-03674-1

**Published:** 2021-12-21

**Authors:** Akshaya Kumar Aliyana, S. K. Naveen Kumar, Pradeep Marimuthu, Aiswarya Baburaj, Michael Adetunji, Terrance Frederick, Praveen Sekhar, Renny Edwin Fernandez

**Affiliations:** 1 Department of Electronics, Mangalore University, Mangalore, India; 2 Rajeev Gandhi College of Engineering and Technology, Puducherry, India; 3 School of Engineering and Computer Science, Washington State University, Vancouver, USA; 4 Department of Engineering, Norfolk State University, Norfolk, USA

**Keywords:** Nanoscience and technology, Graphene, Electronic properties and devices, Electrical and electronic engineering

## Abstract

We report a machine learning approach to accurately correlate the impedance variations in zinc oxide/multi walled carbon nanotube nanocomposite (F-MWCNT/ZnO-NFs) to NH_4_^+^ ions concentrations. Impedance response of F-MWCNT/ZnO-NFs nanocomposites with varying ZnO:MWCNT compositions were evaluated for its sensitivity and selectivity to NH_4_^+^ ions in the presence of structurally similar analytes. A decision-making model was built, trained and tested using important features of the impedance response of F-MWCNT/ZnO-NF to varying NH_4_^+^ concentrations. Different algorithms such as kNN, random forest, neural network, Naïve Bayes and logistic regression are compared and discussed. ML analysis have led to identify the most prominent features of an impedance spectrum that can be used as the ML predictors to estimate the real concentration of NH_4_^+^ ion levels. The proposed NH_4_^+^ sensor along with the decision-making model can identify and operate at specific operating frequencies to continuously collect the most relevant information from a system.

## Introduction

Metal oxides-based sensors with excellent response time and longevity, are candidates for the next generation real-time sensing. Analytical signals from a chemical sensor are dominated by the target concentration. Besides the target molecule, interfering ions, process parameters like solution pH, conductivity, temperature, electrode parameters such as geometry, material properties also contribute to the sensor signal^1–4^. In a dynamic setting, these parameters contribute heavily to the signal erraticism^5^. Response of such intricate systems cannot be interpreted using calibration curves or modeled using mathematical equations.

Metal oxide active layers have highly tunable electrical, optical and mechanical properties^6–9^. Metal oxide nanocomposites with superior material properties and enhanced sensing range has been developed using ZnO, TiO_2_, SiO_2,_ NiO_2,_ Fe_2_O_3_ and CuO^10,11^. Metal electrodes coated with metal oxide ion selective active layers have been used for electrochemical sensing of Ammonium (NH_4_^+^)^12–14^. The enhanced sensitivity of the ZnO is attributed to its surface to volume ratio, electrical conductivity, fast response, wide band-gap (3.37 eV)^15^, large exciton binding energy (60 meV)^16^, and piezoelectricity^17^. Metal oxide functionalization modulates the physicochemical properties of MWCNT thus increasing their ease of dispersion, manipulation, and process ability. Conjugating MWCNT with ZnO have shown to reduce the resistance of the sensing material and metal oxide controls the sensing properties^18–19^. Sensitivity of the electrodes are influenced by the chemical composition and microstructure morphology of the ZnO/MWCNT active layer^16,20,21^. After repeated measurements, metal-oxide integrity is degraded, resulting in a gradual decrease in maximum impedance ($\left|{\mathrm{Z}}_{\mathrm{max}}\right|$) values over the period of time^22^.

### Machine learning aided sensing

Authors have applied machine learning (ML) models to interpret the impedance response of a ZnO/MWCNT NH_4_^+^ sensor, mainly to compensate for the instability and drift in $\left|{\mathrm{Z}}_{\mathrm{max}}\right|$ values. The proposed ML approach centers the estimation of NH_4_^+^ on several predictors using advanced multivariate mathematical models. ML approach is adopted to correlate the impedance changes of a ZnO/MWCNT active layer to NH_4_^+^ concentrations at varying solution conditions. Machine learning aided impedance sensors have been previously reported to classify *Escherichia coli* (*E. coli*) strains JM109, DH5-α, and *Salmonella typhimurium*^23^. However, no study has been reported on machine learning aided impedance sensors for detecting chemicals.

When trying to electrochemically analyze complex mixtures of structurally similar analytes in real matrices, various interferences occur, leading to faulty estimation of target concentration. Instability in electrochemical sensing is caused by structurally similar molecules/ions (Ca^2+^, Mg^2+^, K^+^, H^+^ and Na^+^)^24^, solution pH, sensor fouling, temperature etc.^25^. Application of machine learning techniques in chemical sensing provide several benefits: (1) can cut down sensor optimization cost and time (2) provide unexpected insights into the experimental data (3) can predict the outcome by deciphering non-linear analytical input signals without mathematical fitting (4) can be readily integrated into a IoT setting. Machine learning powered intelligent electrochemical sensors have better accuracy and reliability^26^. Systematic ML investigation discovers the hidden relationship between the analyte sample parameters and sensing signals through data compression, elimination of baseline drifts, normalization, transformations, and so forth^27^.

Various ML models are used to train and extract significant features^28,29^. Support vector machine (SVM) model, used in cancer and pathogen detection, transforms predictor data into higher dimensions in order to maximize the response between the training patterns and the decision boundary^29,30^. The k-Nearest Neighbor (kNN) algorithm has been applied to solve classification problems in biology^31^. Naive Bayes (NB) is a probabilistic classifier based on Bayes theorem with strong (Naive) independence assumptions^32^. Decision tree (DT) based models have been proved to be efficient in various ML applications in particular chlorophyll (Chl) level monitoring^33^, food quality assessment^34,35^ and antioxidants detection in biodiesel^36^. Each node of DT stands for a feature in an instance to be tested, each branch stands for a value that the node can assume, and each leaf stands for a probability density value distribution. Extensively used ensemble ML model is random forest (RF), operates by developing a number of DTs for classification and regression with addressing overfitting issues^37,38^. Artificial neural networks (ANN) are primarily used to deal with the non-linear problems^39^. These features are particularly beneficial for real-time operations and quick decisions.

Most sensing systems either rely on an equivalent circuit^40^ or a deterministic model^41^ to make predictions about the anticipated output concentrations. Often, the models have stringent boundary conditions and require extracting sub features to correlate to calibration curves. We hypothesize that a machine learning aided analytical sensing will enhance the accuracy of field deployable sensors operating at dynamic experimental conditions. The success of machine learning aided sensors depend on extracting the right features from an electronic signal or a frequency response. Signals that appear random or decipherable only using complex mathematical equations often have repeatable features that have a collective value after preprocessing^42^. The extracted features are often either peak of waveforms, the rise and fall rates, distance between peaks etc. Impedance spectrum of the sensor provides a fingerprint for a measured sample.

In this paper, we explain how the sensing accuracy of a metal-oxide based impedance sensing system can be improved using a machine learning approach that identifies predominant spectral features to predict unknown NH_4_^+^ concentrations. We have analyzed the performance of in-house fabricated F-MWCNT/ZnO-NF nanocomposite sensors with varying ZnO:MWCNT ratios. The impedance characteristics of the sensors to varying NH_4_^+^ concentrations were analyzed to extract impedance and frequency features. Datasets that capture the uniqueness of an impedance response were generated along with experimental parameters, interfering ion concentrations. Features like $\left|{\mathrm{Z}}_{\mathrm{max}}\right|$, $\left|\mathrm{Z}\right|$ at fixed frequencies, and average slope of the impedance curve were found to be important predictors. ML models like kNN, random forest, neural network, Naïve Bayes and logistic regression model were trained using datasets with more than 18 predictors. Some of the predictors were transformed to closely resemble a normal distribution as part of the preprocessing. The most dominant predictors were identified using feature importance analysis.

## Experimental and methods

### Chemicals

**Figure 1:**
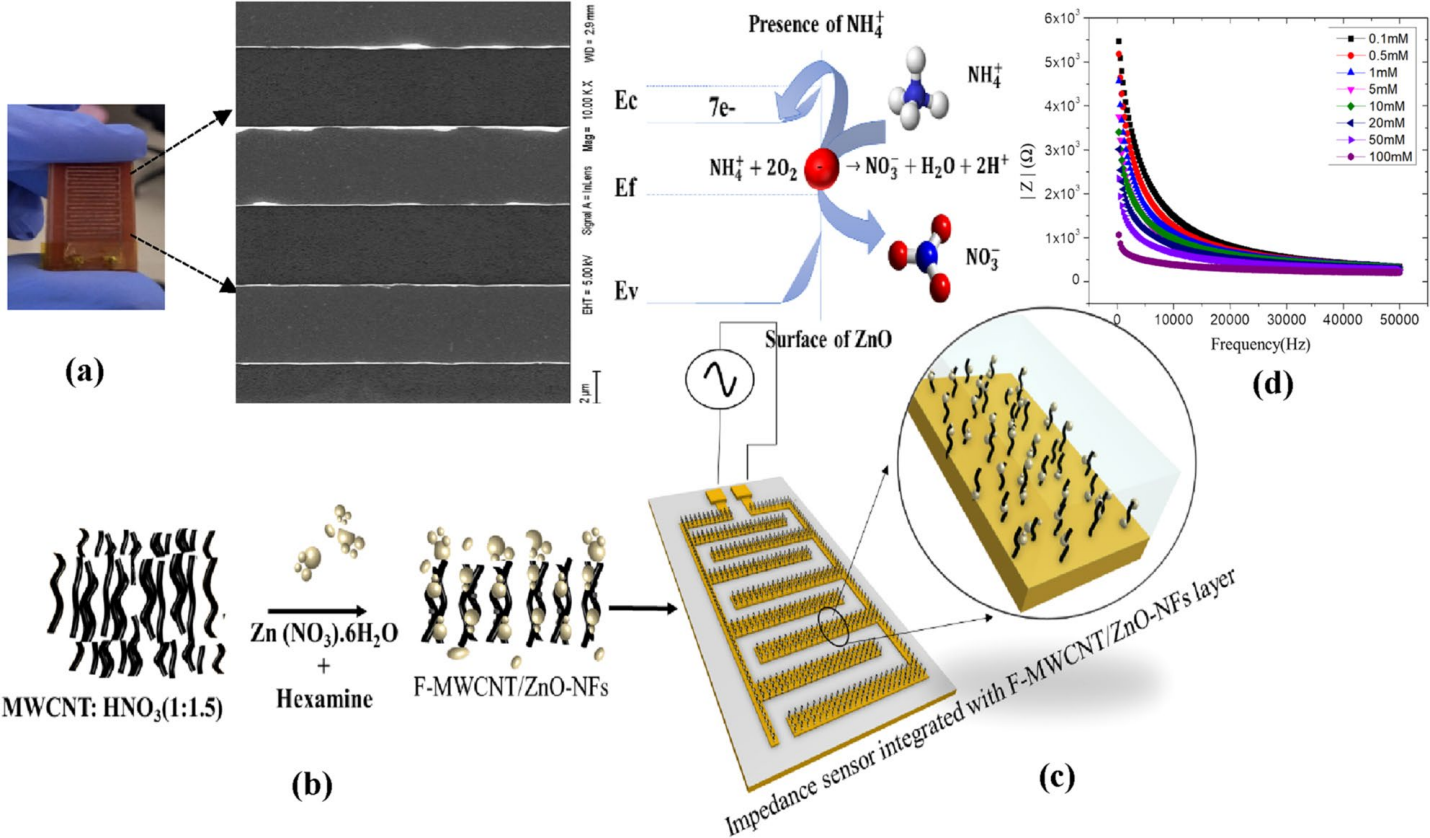
(**a**) Screen printed interdigitated electrodes (IDEs), (**b**) F-MWCNT/ZnO-NFs nanocomposites are synthesized by functionalizing MWCNT using ZnO nanoparticles. (**c**) NH_4_^+^ selective impedance sensors are fabricated by embedding F-MWCNT/ZnO-NF active layers on interdigitated arrays and (**d**) electrochemical response of the NH_4_^+^ selective impedance sensors.

Multi-Wall Carbon nanotube (MWCNT), Zinc nitrate hexahydrate (H_12_N_2_O_12_Zn), Sodium hydroxide (NaOH), Hexamine((CH_2_)_6_N_4_), Copper (Cu), Fibre epoxy resins, Di-methyl sulfoxide (DMSO), Nitric acid (HNO_3_), Ammonium hydroxide (NH_4_^+^OH) and Ethanol are the precursor materials and solvents used to formulate the F-MWCNT/ZnO-NFs nanocomposites. The precursor materials and analytes were purchased from Sigma Aldrich. Interdigitated electrode structures were made from Copper (Cu) and Fiber epoxy resin (Fig. 1c).

### Device characterization

The surface orientation of the F-MWCNT/ZnO structure is studied using field emission scanning electron microscopy (FeSEM) with energy-dispersive X-ray spectroscopy (EDS). Crystal phase and orientation of F-MWCNT, ZnO and F-MWCNT/ZnO nanocomposites were examined using an X-ray diffractometer (XRD). Further, electrochemical performance of NH_4_^+^ detection device was measured using an Agilent 4294A precision impedance analyzer system. Measurements were repeated and analyzed using multiple sets of sensors.

### Fabrication of F-MWCNT/ZnO-NFs nanocomposites based NH_4_^+^ smart sensor

The fabrication process of screen-printed IDEs modified with F-MWCNT/ZnO active layer is illustrated in Fig. 1. The Copper (Cu) based IDEs structures were screen printed on the fiber epoxy substrate as described in our previous publication^16^. The CIRCAD software-oriented IDE’s design consist of total 18 fingers and provide a total sensing area of 18 × 15 mm^2^ for the NH_4_^+^ analytes.

F-MWCNT was functionalized using ZnO nanoparticles to form F-MWCNT/ZnO-NFs nanoflowers (NFs) structures. MWCNT is added with Nitric acid (HNO_3_) in the ratio of 1:1.5 solution is made homogeneous. The obtained solution is further refluxed for 8 h at 110 °C, consequently F-MWCNT powder treated with 0.1, 0.3 and 0.5 M Zn (NO_3_)·6H_2_O to get the ZnO in the proportion of 1%, 3% and 5%. Three variants of Nanocomposites were synthesized by varying the MWCNT: ZnO ratio. The binding agent (0.01 M Hexamine) is added to the respective homogeneous nanocomposite solutions to create three types of sensors (S1, S2 and S3) with varying ZNO:MWCNT ratio. The mixture was refluxed for 3 h at 120 °C and precipitate is dried at 80 °C for 10 h to get the F-MWCNT/ZnO-NFs composites. The synthesized products were investigated in terms of morphological and structural properties. Nanocomposites further dispersed in the Di-methyl sulfoxide (DMSO) solvent were drop casted over the screen-printed IDEs surface.

## Results and discussion

### Characterizations of IDEs modified F-MWCNT/ZnO nanocomposite active layer

**Figure 2:**
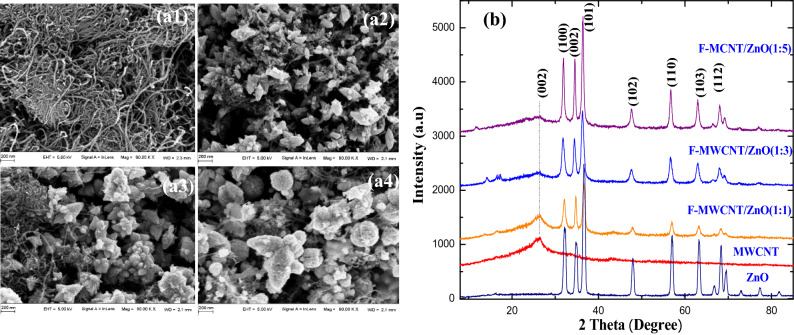
(**a**) FeSEM images of F-MWCNT (**a1**), F-MWCNT/ZnO-NFs composites with proportion of 1:1 (**a2**), 1:3 (**a3**), 1:5 (**a4**) and (**b**) X-ray diffraction (XRD) spectrum for ZnO, F-MWCNT, F-MWCNT/ZnO-NFs.

The layout and prototype of the screen-printed IDEs and FeSEM images of the electrode patterns are shown in Fig. 1c. The width and space between the two electrodes designed for 500 μm which comes to ≈ 460 μm after printing. Evident from the field emission microscopy (FeSEM) studies on F-MWCNT (Fig. 2a1), F-MWCNT/ ZnO-NFs composites with proportion of 1:1 (Fig. 2a2), 1:3 (Fig. 2a3) and 1:5 (Fig. 2a4), the morphology of F-MWCNT/ZnO-NFs composites with varying ZnO percentages were distinctly different. Formation of nanoflowers (NF) inclusions over the F-MWCNT were observed. The majority of the MWCNT and F-MWCNT particles are uniformly distributed in high compactness with almost parallel alignment to the surface. Nanoflowers (NFs) are assembled in bunches which contain sub-NFs. Further characterization of the NFs attributed a hexagonal structure to NFs confirming good crystallinity. We noticed that ZnO percentage in the composite defines the orientation of the NFs in such a way that its branching and petal size is proportional to the ratio of ZnO (1 to 5%).

Morphology and chemical composition of the nanocomposites were investigated using Energy Dispersive Spectroscopy (EDS) (Fig. S2). To analyze the crystalline nature of the particles, X-ray diffraction (XRD) study for ZnO, F-MWCNT, F-MWCNT/ZnO-NFs (S1, S2 and S3) samples were done and pattern is plotted in Fig. 2b. Obtained pattern shows maximum intensity for the peak positioned at 2θ = 36.56° for ZnO along the (101) plane and relates the growth along c-axis parallel to the substrate.

### Evaluation of electrochemical sensing performance of F-MWCNT/ZnO-NFs composites active layer

Impedance response of the F-MWCNT/ZnO-NFs composite samples were recorded using Agilent 4294A precision impedance analyzer. The performance of the sensors was evaluated using standard NH_4_^+^ solutions (1–100 mM). Enhanced sensitivity and stability of the F-MWCNT/ZnO-NFs is attributed to the higher surface area and direct electron mobility^11^. NH_4_^+^ ions in the immediate vicinity of ZnO-NF active layers, are converted into NO_3_^−^^6,43^. NH_4_^+^ analytes adsorbed on ZnO-NFs are nitrified causing a decrease in the overall impedance of the sensor which induces a change in the local electrical fields and a proportional change in the electrical properties of the F-MWCNT/ZnO-NFs nanocomposites. The surface of ZnO-NFs will react with oxidizing and reducing analytes due to the presence of adsorbed oxygen species on the outermost layer, which subsequently gets ionized into O^2−^, O^−^ or O_2_^−^.

Three types of the F-MWCNT/ZnO-NF nanocomposite sensors were constructed by varying the ZnO:MWCNT weight percentages. Sensors S1, S2 and S3 had ZnO:MWCNT ratios of 1:1, 1:3 and 1:5 respectively. Impedance spectra of the sensors showed an inversely proportional response with frequency in the 1–10 kHz range (Fig. S3), clearly at higher frequencies the total impedance of the sensor decreased^44,45^. Higher $\left|{\mathrm{Z}}_{\mathrm{max}}\right|$ were observed for S3 (1:5) compared to S1 (1:1) and S2 (1:3). Results confirm the predominant effect of ZnO weight percentages on the impedance characteristics of F-MWCNT/ZnO-NFs composites. $\left|{\mathrm{Z}}_{\mathrm{max}}\right|$ values also tend to slightly vary from device to device. Such anomalies lead to inaccurate analysis of target concentrations.

### Ammonium sensing

**Figure 3:**
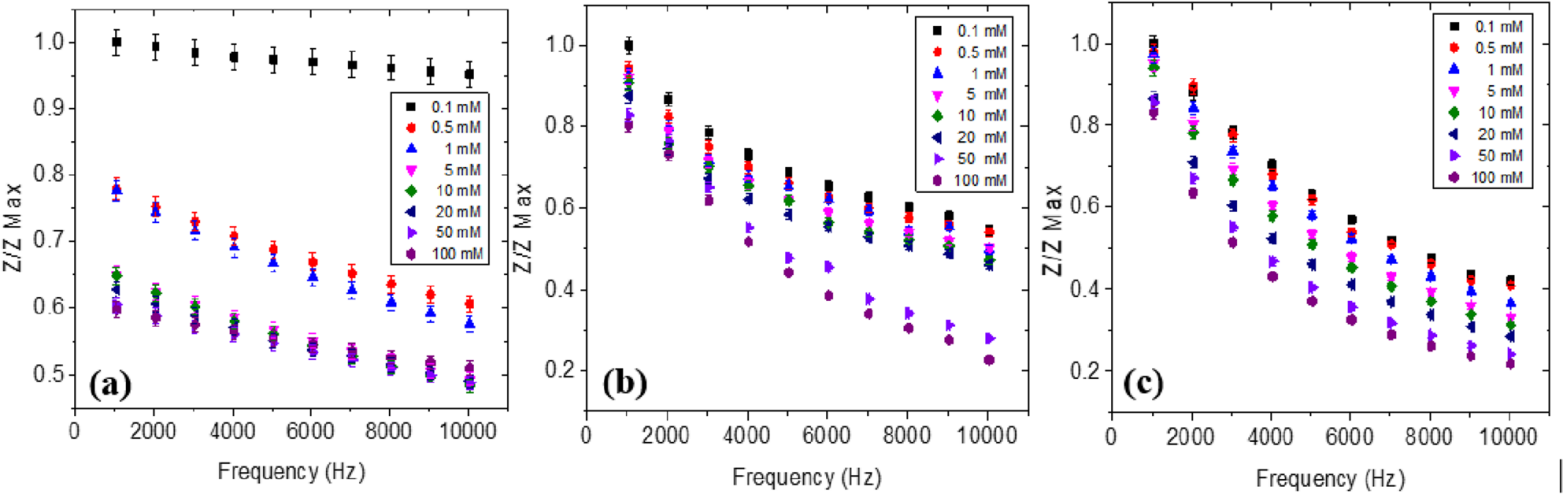
Impedance (Z/Z_max_) versus frequency response of the sensors (**a**) S1, (**b**) S2 and (**c**) S3 at 0.1–100 mM NH_4_^+^ ion concentrations.

The performance of the sensors S1, S2 and S3 was evaluated using standard NH_4_^+^ solutions (1–100 mM) (Fig. 3). The presence of NH_4_^+^ molecule in the dielectric (liquid medium) causes a change in the sensor impedance. A proportional decrease in the impedance magnitude was observed with increasing frequency (1 to 10 kHz) in all the three sample conditions. Linearity of the response was found to decrease with frequency. It was observed that response of impedance magnitude is influenced by the pH environment of the NH_4_^+^ testing samples. The impedance sensitivity of the electrode was drastically affected by the pH environments, the response being acute in the 1–10 kHz range.

**Figure 4:**
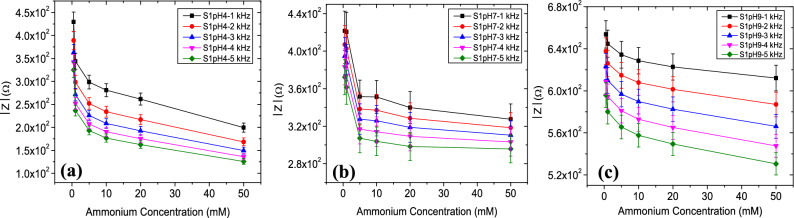
Impedance magnitude (Ω) versus NH_4_^+^ ion concentration (0.5–50 mM) response of the sensor S1 in the (**a**) acid, (**b**) neutral and (**c**) alkaline sample conditions.

**Figure 5:**
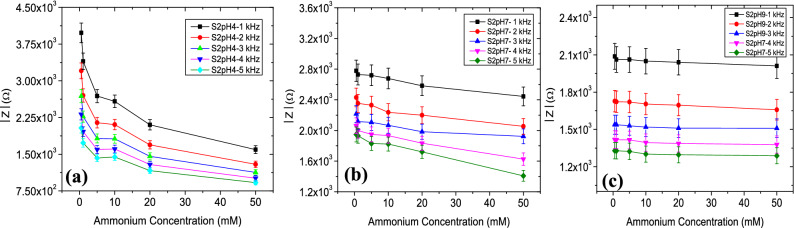
Impedance magnitude (Ω) versus NH_4_^+^ ion concentration (0.5–50 mM) response of the sensor S2 in the (**a**) acid, (**b**) neutral and (**c**) alkaline sample conditions.

**Figure 6:**
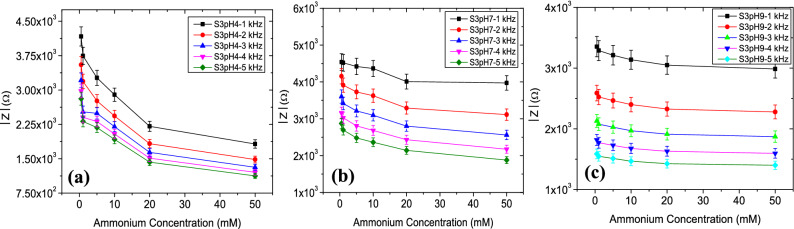
Impedance magnitude (Ω) versus NH_4_^+^ ion concentration (0.5–50 mM) response of the sensor S3 in the (**a**) acid, (**b**) neutral and (**c**) alkaline sample conditions.

Results indicate that higher pH conditions cause an enhanced impedance value for S1, S2 and S3. It can be noted that impedance values of all sensors decreased with increasing NH_4_^+^ levels in acid (Figs. 4a, 5a, 6a), neutral (Figs. 4b, 5b, 6b) and alkaline (Figs. 4c, 5c, 6c) conditions. The device sensitivity to NH_4_^+^ was also found to vary with pH. Critical values such as the $\left|{\mathrm{Z}}_{\mathrm{max}}\right|$ and the frequency dependency of $\left|\mathrm{Z}\right|$ were found to vary from device to device. $\left|{\mathrm{Z}}_{\mathrm{max}}\right|$ values for sensor S3 operated at pH 4 was ~ 10.5% higher when operated in pH 9 and its ~ 23.8% higher for sensor S2. $\left|{\mathrm{Z}}_{\mathrm{max}}\right|$ values were comparatively stable for S1 sensors with a variation of ~ 2%. The rate of change of $\left|{\mathrm{Z}}_{\mathrm{max}}\right|$ is highest for pH 4 whereas increase in Z_max_ was very gradual at pH 7 and 9.

**Figure 7:**
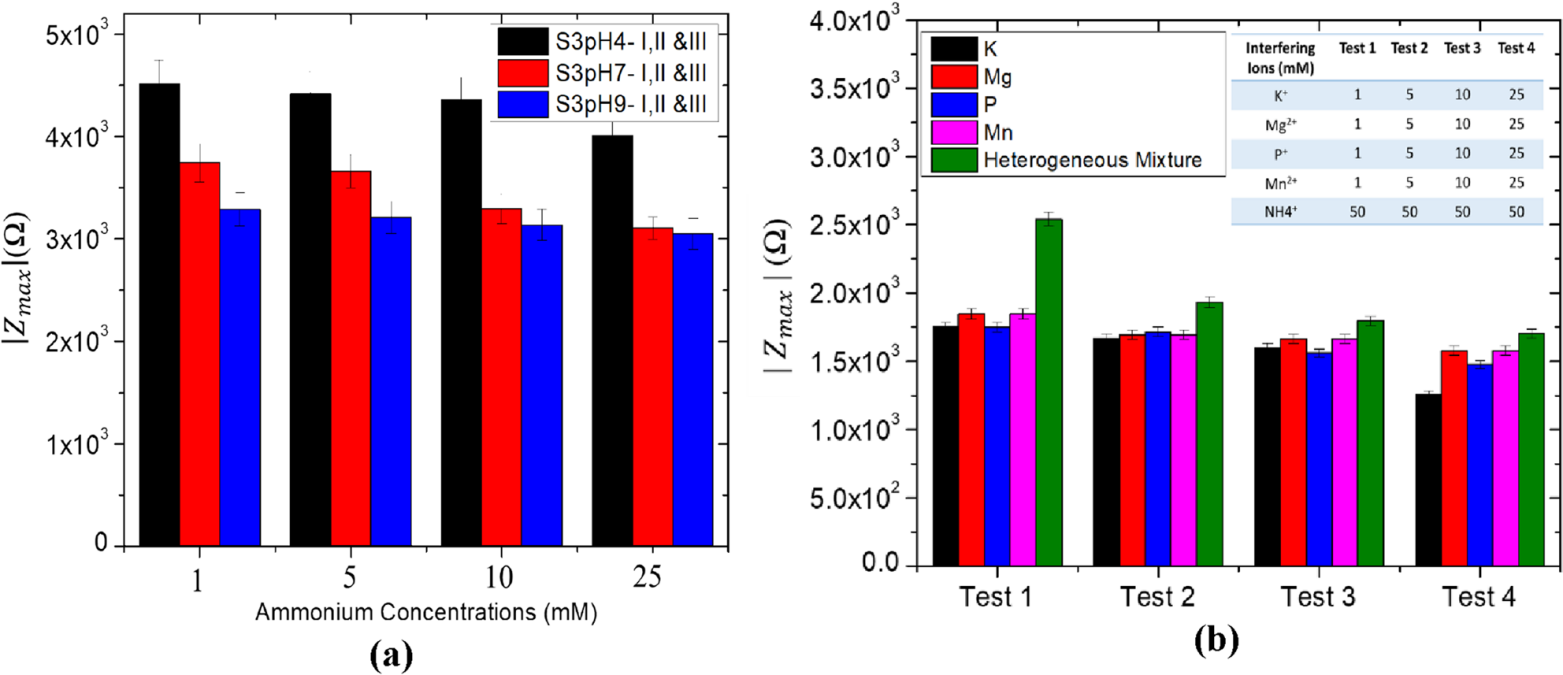
(**a**) $\left|{\text{Z}}_{\text{max}}\right|$ versus NH_4_^+^ concentration (1–20 mM) for three similar set of S3 devices in acid, neutral and alkaline pH conditions. (**b**) Selectivity performance of the sensor in the presence of Interfering ions (K^+^, Mg^2+^, P^+^, Mn^2+^ and Heterogeneous mixture) concentrations levels.

Figure 7a shows the response of $\left|{\mathrm{Z}}_{\mathrm{max}}\right|$ versus NH_4_^+^ concentration (1–20 mM) for three S3 devices in the acid, neutral and alkaline sample conditions. $\left|{\mathrm{Z}}_{\mathrm{max}}\right|$ values can be seen to decrease consistently with increasing NH_4_^+^ concentrations. $\left|{\mathrm{Z}}_{\mathrm{max}}\right|$ values for S3 I, II and III sensors showed ~ 5% variation which can be due to variation in the active layer thickness among the sensor prototypes. Response of the sensors were interpreted using linear impedance versus NH_4_^+^ calibration plots (Figs. S7, S8 and S9). The correlation coefficient (R^2^) of these plots is in range of 0.75–0.90. Sensitivity of the sensors were between 76.09 and 1.06 mM/Ω with an average correlation coefficient (R^2^) of 0.81. A decrease in sensitivity was observed when the solution pH was varied from acid to alkaline. This can be attributed to the non-uniformity in the distribution of ZnO-NFs on electrode active area (Fig. 2a). To interpret the stability of the sensor, impedance characteristics were recorded every 500 s for a total time duration of 4000 s and further measured every day for 7 continuous days. The results confirm the stable performance of the fabricated device.

To evaluate the selectivity of the NH_4_^+^ sensor, we introduced 1–25 mM of interfering ions (K^+^, Mg^2+^_,_ P^+^, and Mn^2+^) in 50 mM NH_4_^+^ sample. Figure 7b reveals the selectivity performance of the device in the presence of interfering ions. Four experiments (Test 1–4) were performed to analyze the effect of interfering ions. Test 1–4 was conducted in the presence of 1, 10 and 25 mM each of the interfering ions (K^+^, Mg^2+^, P^+^, and Mn^2+^). Experiments were also conducted using a heterogenous mixture of all the interfering ions. In the presence of 1–25 mM interfering ions with 50 mM NH_4_^+^, $\left|{\text{Z}}_{\text{max}}\right|$ values were found to change sigificantly in the presence of interfering ions. A drift of ~ 5.14 to ~ 23.15% in $\left|{\text{Z}}_{\text{max}}\right|$ was observed in NH_4_^+^ sensor due to chemisorption of interfering ions in the MWCNT/ZnO active layer. Heterogenous mixture of ions resulted in a significant variation of ~ 4.5% (Fig. 7b) in the $\left|{\text{Z}}_{\text{max}}\right|$ values.

### Machine learning based target prediction

**Figure 8:**
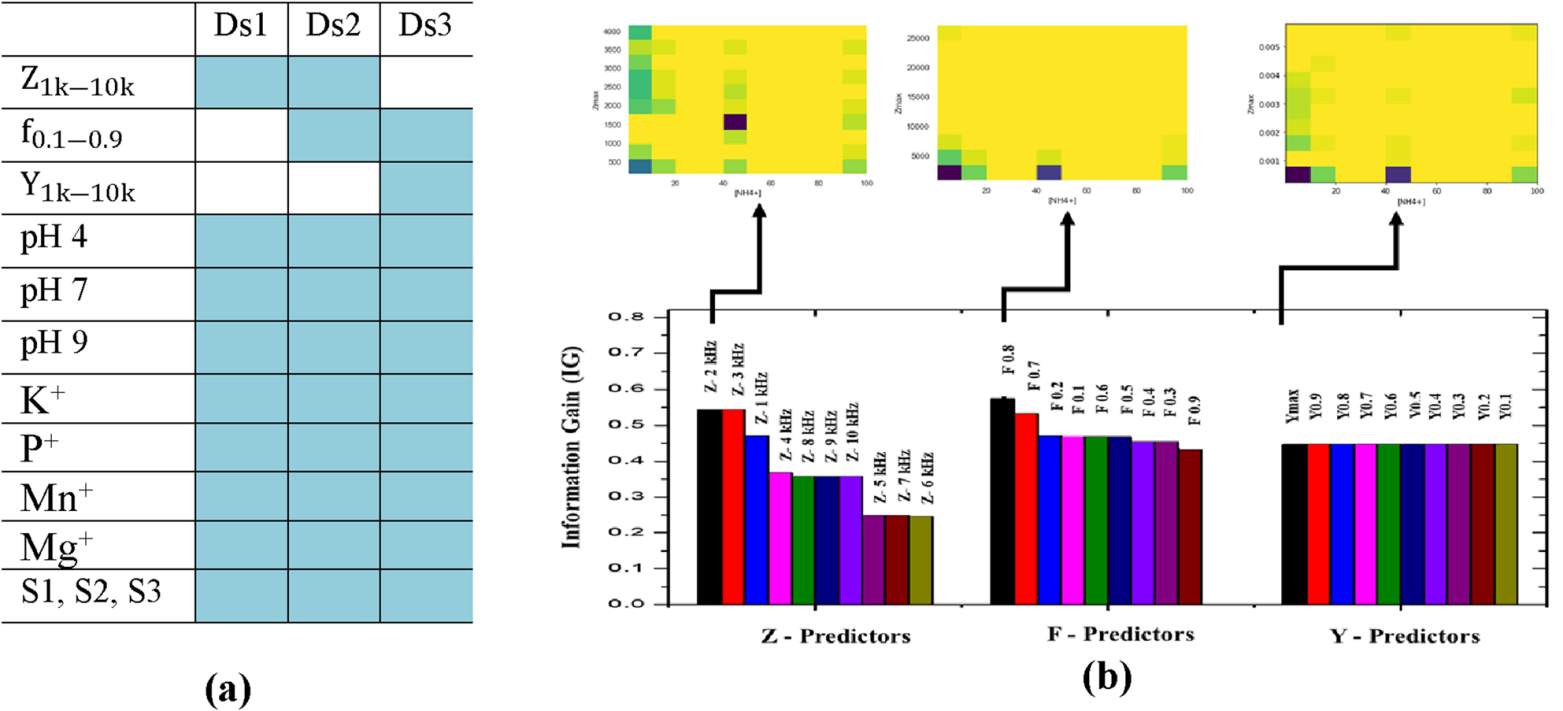
(**a**) Impedance spectral features and experimental parameters used as predictors in various datasets. (**b**) Information gain scores for Z, F and Y predictors. Insets show the variation in ${\mathrm{Z}}_{\mathrm{max}}$ values of predictors with maximum IG scores (${\mathrm{Z}}_{2\mathrm{k}}$, ${\mathrm{f}}_{0.8}, {\mathrm{Y}}_{\mathrm{max}}$).

Prediction of target variable concentration (NH_4_^+^) was done using 28 predictors in which 8 are experimental features and 20 are impedance and frequency features of the Frequency-impedance (F-Z) spectra (Fig. 8). S1, S2 and S3 are categorical predictors that indicate the type of sensor. The extracted features of the spectra are stored in a structure with a sensor and session ID number encoded in CSV file for each impedance spectra. Missing values were replaced with the median value of the predictor. 80% of dataset is assigned as training data set and the other 20% were assigned for testing. The accuracy of the ML regression models was analyzed using a confusion matrix in order to compare the predicted and true values. ML analyses were performed using the Orange toolbox by writing Python scripts accessing the Orange API. Python Script widget are used to run python scripts in Orange to perform additional functionalities like feature importance^46^. The updated variables from the Python script are used as outputs of the Python Script widget.

### Feature extraction and model training

**Figure 9:**
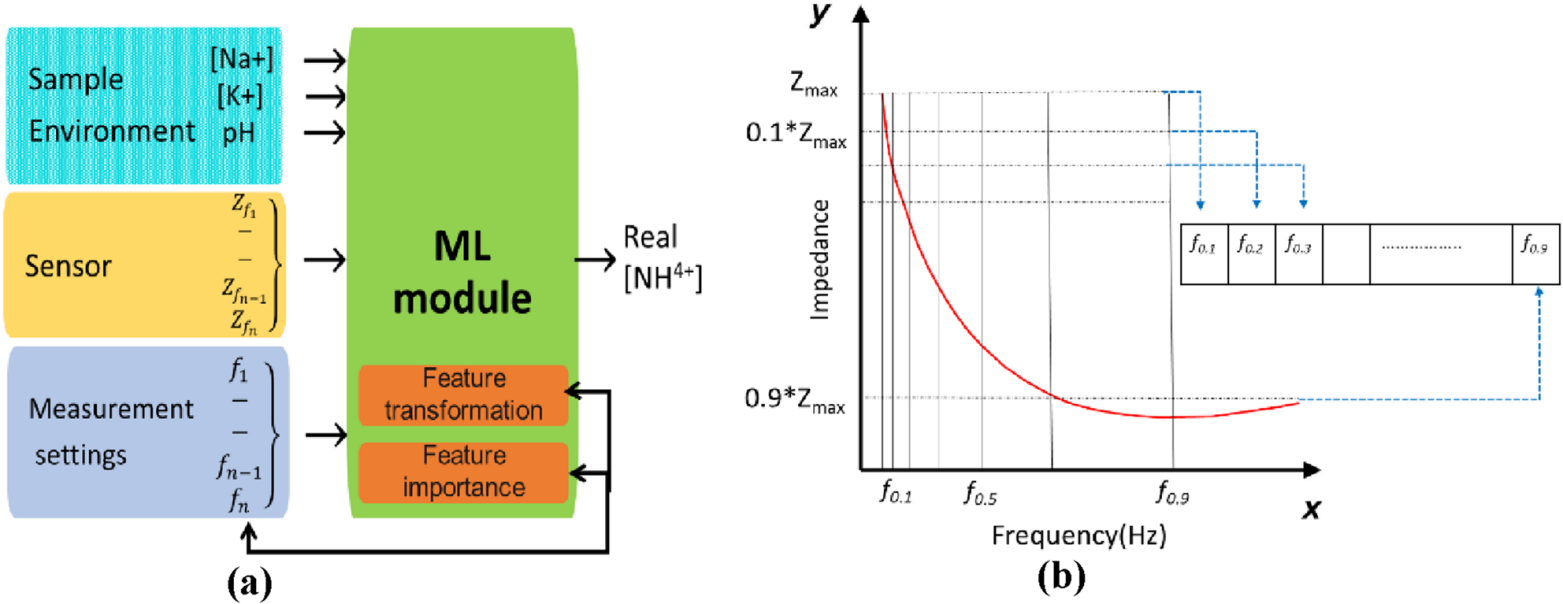
(**a**) Predictors of the ML model are extracted from Impedance Spectral features, sample parameters, and experimental settings. (**b**) Predictors ${\mathrm{f}}_{0.1}$ to ${\mathrm{f}}_{0.9}$ are the frequencies at which the impedance values are 0.1–0.9 times of ${\mathrm{Z}}_{\mathrm{max}}$.

Predictors of our model were extracted from the impedance spectra, sample features, and experimental settings (Fig. 9). Both impedance magnitude and frequency features are used as predictors to capture the dynamics of the original spectra. Individual features were expanded into multiple predictors in order to better represent a non-linear input–output relationship thereby eliminating outliers and reducing computational time^47^. The frequencies at which a fixed change in impedance occurs are taken as the derivative frequency values (${\mathrm{f}}_{\mathrm{n}}$) of a spectrum (Fig. 9b). Predictors ${\mathrm{f}}_{0.1}$ to ${\mathrm{f}}_{0.9}$ are the frequencies at which the impedance values are 0.1–0.9 times of ${\mathrm{Z}}_{\mathrm{max}}$ from the impedance spectra, we extracted derivative frequencies ${\mathrm{f}}_{\mathrm{n}}$ at $${\mathrm{Z}}_{\mathrm{n}}=\mathrm{n}*{\mathrm{Z}}_{\mathrm{max}} \left\{\begin{array}{c} 0.1\le {\text{n}}\le 0.9\}\end{array}\right.$$. We hypothesize that representing the impedance variations as ${\mathrm{f}}_{\mathrm{n}}$ will reduce the dependency of the spectrum on electrode area. ${\mathrm{f}}_{\mathrm{n}}$ starts to increase at the onset of depletion and a maximum value of ${\mathrm{f}}_{\mathrm{n}}$ is observed at $0.9*{\mathrm{Z}}_{\mathrm{max}}.$ Although ${\mathrm{Z}}_{\mathrm{max}}$ value is a function of the area of the electrode, the frequency response is influenced by the dielectric property of the material and the environment. Total surface area of an electrode may decrease after cycles of repeated measurements as the nanocomposite structures in the active layer tend to deteriorate.

We transformed the impedance features into admittance values in order to improve the model. Raw impedance data were preprocessed using a running mean prior to modeling. But excess data smoothing showed a tendency to eliminate important nonlinear patterns. Hence, we adopted a Box-Cox transformation method that uses separate estimation procedures to transform the impedance (Z) data so that it closely resembles a normal distribution. A Box-Cox transformation was used to estimate the transformation.

$${\mathrm{x}}^{*}=\left\{\begin{array}{ll}\frac{{\mathrm{x}}^{\uplambda}-1}{\uplambda\, {\mathrm{x}}^{\uplambda -1}},&\quad \lambda \ne 0\\ \tilde{\mathrm{x }}\,\mathrm{log}\,{\text{x}}, &\quad\lambda =0\end{array}\right.$$

The Box-Cox procedure, originally intended as a transformation of a model’s outcome, uses maximum likelihood estimation to estimate a transformation parameter λ in the equation which is estimated from the data. Values of λ map to transformations. When λ = 1 (no transformation), λ = 0 (log), λ = 0.5 (square root), and λ =  − 1 (inverse). For impedance (Z) values, λ was estimated to be − 1 which is effectively the inverse transformation. Hence, we adopted the admittance representation in our datasets using the feature constructor tool in Orange.

### Prediction efficiency of the decision-making model

From the confusion matrices, scoring frameworks such as CA (Accuracy classification score), Precision (precision computation), recall (computation of true positives and false negatives), F1 score (weighted average of precision and recall) and AUC (stored predictions and actual data in model testing) were obtained to evaluate the prediction efficiency of each model. Scoring framework takes the decimal values of probability between 0 and 1. Evident from the Confusion matrices (Tables S5–S10), are the prediction accuracy of all the models among which RF is the most accurate (Table S5). Among all the models that we tested, RF model was the most efficiency in predicting the concentration range, which has a precision score of 0.83–0.89 depending upon the predictor variables. Using impedance only dataset, RF displayed a precision of ~ 0.83. Precision values of the RF model increased from 0.83 to 0.85 when transformed 1/Z values were used. The precision of the RF model was increased further to ~ 0.87 after including derivative frequency ${(\mathrm{f}}_{\mathrm{n}})$ predictors. Accuracy was highest for RF model, when admittance (1/Z) were used as predictor instead of impedance values. Prediction accuracy of the model was lower for samples in low pH solutions. This may be attributed to high variance in Z values in low pH solutions. The accuracy was found to be highest in basic pH.

All other models like ANN and kNN had lower prediction accuracy (< 0.70). SVM models with four different kernels (polynomial, sigmoidal, linear, and radial basis function) were analyzed. However, SVM showed the least accuracy (0.24–0.47) while testing for unknown samples.

### Feature importance estimation for maximum relevance and minimum redundancy criterion

Extraneous predictors erode the performance of a model and also increases computational cost. It is important to identify a subset of predictors with high prevalence. Among the predictors, some of them have a significant influence on the response of the model. Feature importance was tested by permuting the predictors and measuring the decrease in accuracy. In order to identify the predictor combination that is relevant to the response, we made 3 datasets containing predictors derived from the features of the impedance spectra. DS (1) ${\mathrm{Z}}_{1\mathrm{k}-10\mathrm{k}}$, (2) ${\mathrm{Z}}_{1\mathrm{k}-10\mathrm{k}}$, ${\mathrm{f}}_{0.1-0.9}$ (3) ${\mathrm{Y}}_{1\mathrm{k}-10\mathrm{k}}$, ${\mathrm{f}}_{0.1-0.9}$ (Fig. 8a).

A complete list of all features and their Information Gain (IG) scores for RF model is shown in Table 1. IG scores indicate the significance of a predictor in the model. The IG scores of various predictors are ranked based on highest and lowest precedence features. We evaluated the information gain for each variable in order to select only those variables that maximizes the information gain. IG score provides a baseline for comparison; some of the features are removed based on feature importance scores.

**Table 1:**
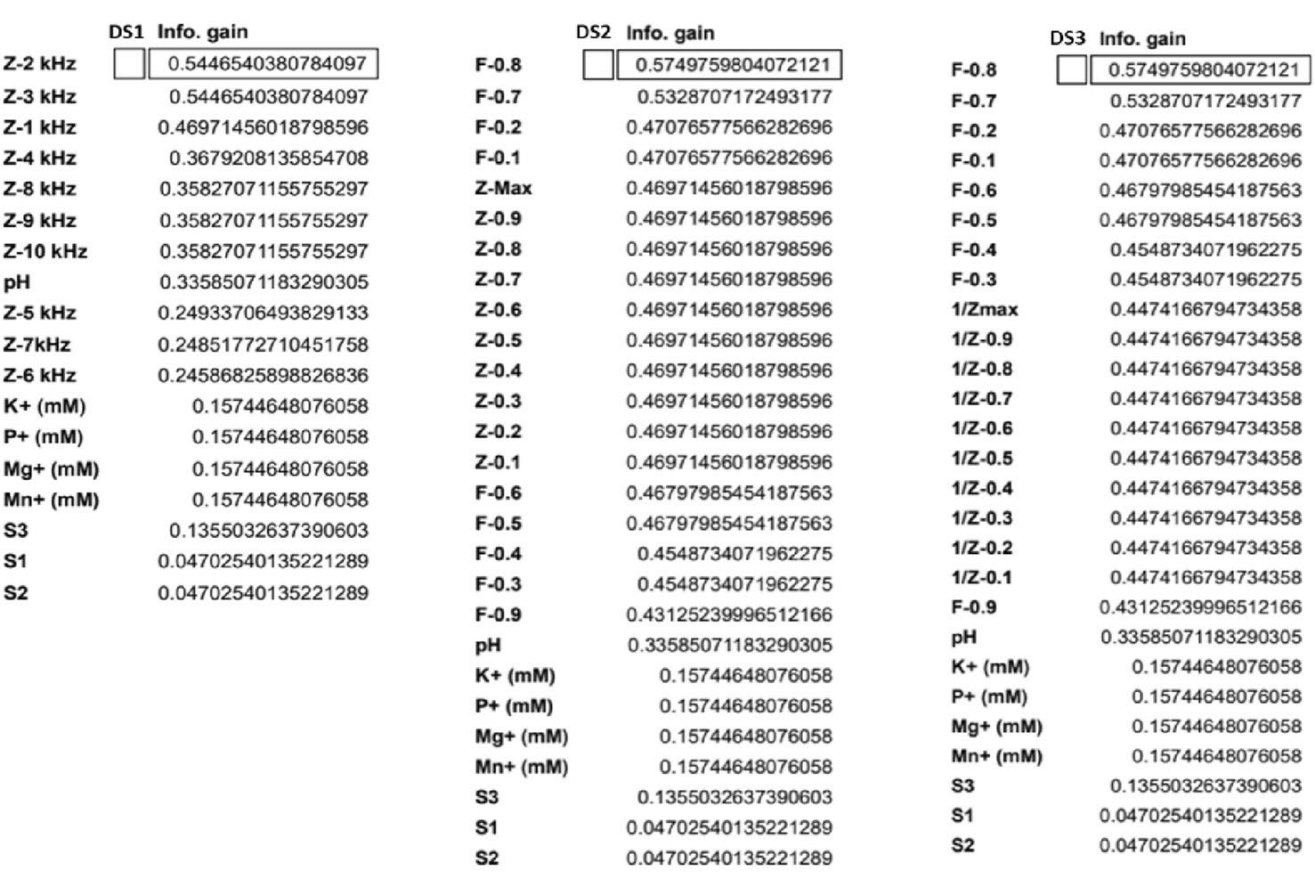
Information gain (IG) scores for DS1, DS2 and DS3.

Among three datasets analyzed, each consisting of ~ 150 data points and 18 features, ${\mathrm{Z}}_{2\mathrm{k}}$, the Z value corresponding to the impedance at 2 kHz, had the highest IG score 0.544 which is higher among all Z predictors (${\mathrm{Z}}_{1\mathrm{k}-10\mathrm{k}}$). IG scores were higher for ${\mathrm{f}}_{\mathrm{n}}$ predictors. Among them ${\mathrm{f}}_{0.8}$ had the highest score (0.574). S predictors, S1, S2, S3, was found to have low info gains (~ 0.047). Following points summarizes our findings from the feature analysis.

1. It was clear from the IG scores that categorical S-predictor parameter S3 has a higher significance than S2 and S1
2. Impedance predictors had higher IG scores than Admittance.
3. Frequency predictors (${\mathrm{f}}_{\mathrm{n}}$) had a higher significance than impedance or Admittance predictors. Among the ${\mathrm{f}}_{\mathrm{n}}$ predictors, ${\mathrm{f}}_{0.8}$ (0.574) had the highest significance and ${\mathrm{f}}_{0.9}$ (0.43) had lowest significance.
4. The effect of interfering ions and pH were consistent across all types of devices (S1, S2 and S3). For all datasets, IG scores for pH was 0.33 and interfering ion concentrations scores were 0.157.

Besides analyzing the impedance spectra for prominent features, our study can be used to design impedance sensing circuits specifically for a sensor. The model is capable of identifying and operating at specific sensing frequencies to continuously collect the most relevant information from a system.

## Conclusions

We report a smart NH_4_^+^ sensing system based on F-MWCNT/ZnO-NFs sensor and a ML model for the purpose of accurately correlating sensor impedance to NH_4_^+^ concentrations. F-MWCNT/ZnO-NFs composites with varying ZnO:MWCNT ratio (S1, S2, and S3) were analyzed for their NH_4_^+^ sensitivity. The machine learning based decision support tool which is the new frame of reference has been designed to interpret the features of impedance spectra and predict the real NH_4_^+^ concentrations by mitigating the effects of non-linearity and impedance drifts. ML analysis has led to identification of dominant predictors of our sensing system. Our work has confirmed the possibility of integrating a custom ML module in an impedance sensing system in order to mitigate the detrimental effect of interference ions and pH on sensitivity and selectivity of F-MWCNT/ZnO-NF active layers. Different models such as kNN, random forest, neural network, Naïve Bayes and logistic regression support have been contrasted and discussed. Depending on the analyte concentration and the metal-oxide, the proposed model can be extended to most impedance-based sensing systems, although dominant features have to be reanalyzed using feature scores and feature transformation.

## Supplementary Information

Supplementary Information.
