# Supplementary material for: Machine learning-assisted ammonium detection using zinc oxide/multi-walled carbon nanotube composite based impedance sensors
Source: Sci Rep. 2021 Dec 21;11:24321. doi: 10.1038/s41598-021-03674-1 (PMC8692315; doi:10.1038/s41598-021-03674-1)
Supplement: Supplementary file 1 — Supplementary Information. [file 41598_2021_3674_MOESM1_ESM.pdf]

Supplementary materials

Synthesis process of F-MWCNT/ZnO-NFs composites:

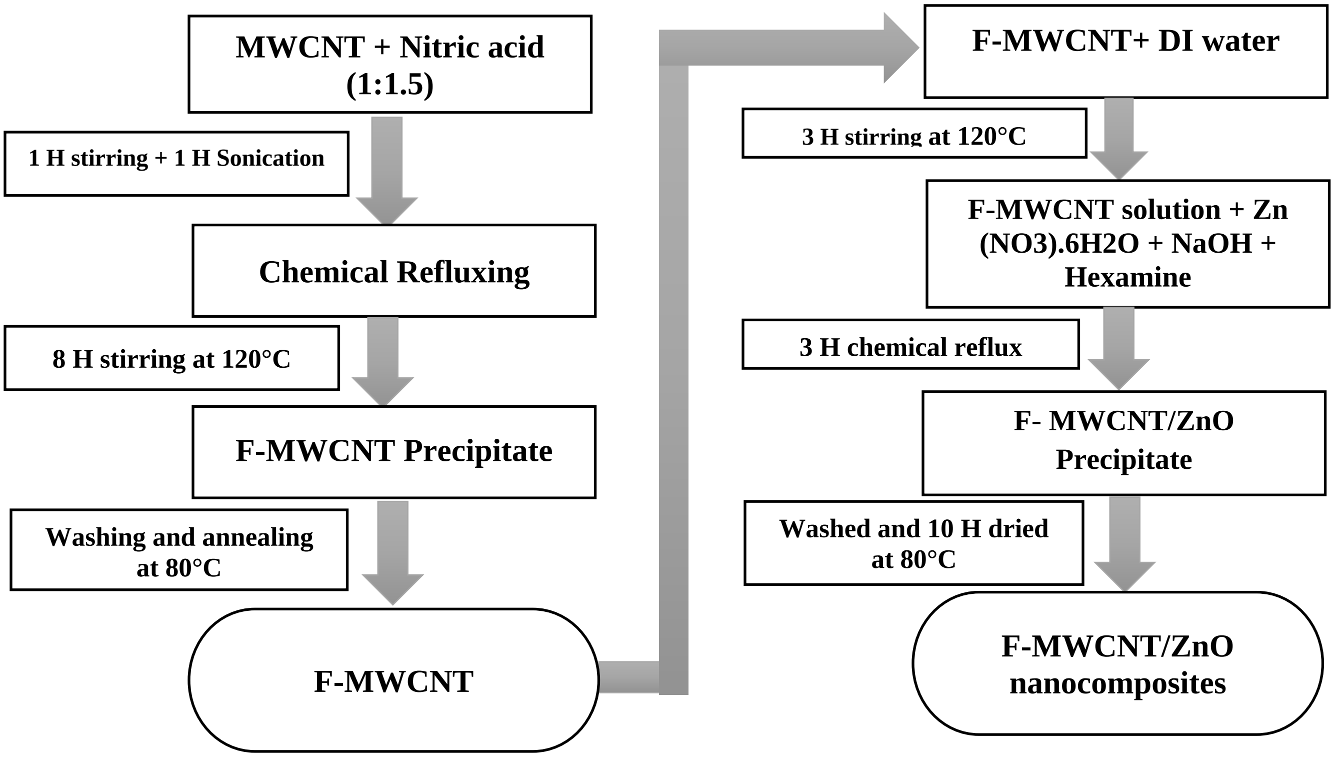

Flow chart. S1 Synthesis process of F-MWCNT/ZnO-NFs composites

Electrochemical sesnor measurement setup:

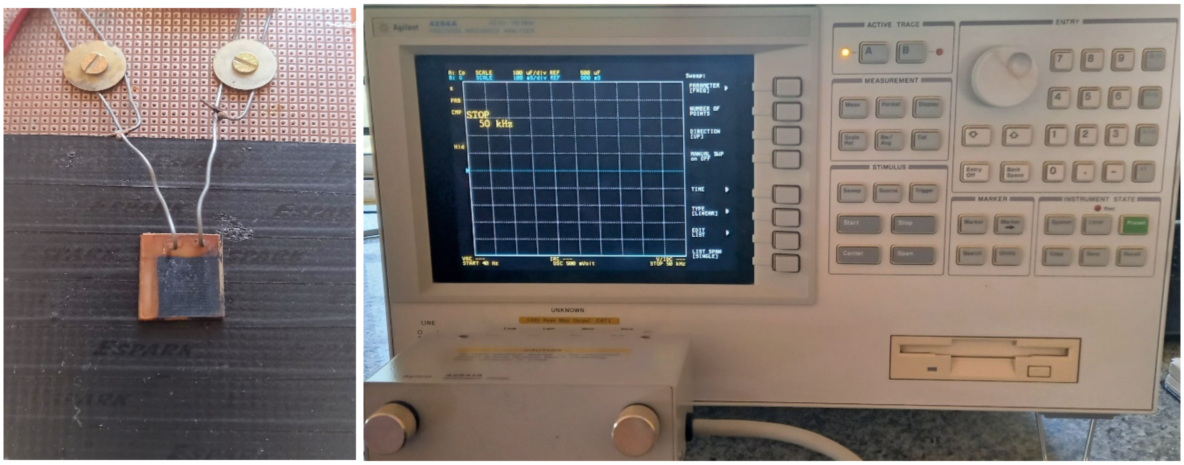

Fig. S1(a) Electrochemical measurement setup of Agilent 4294A precision impedance analyzer system

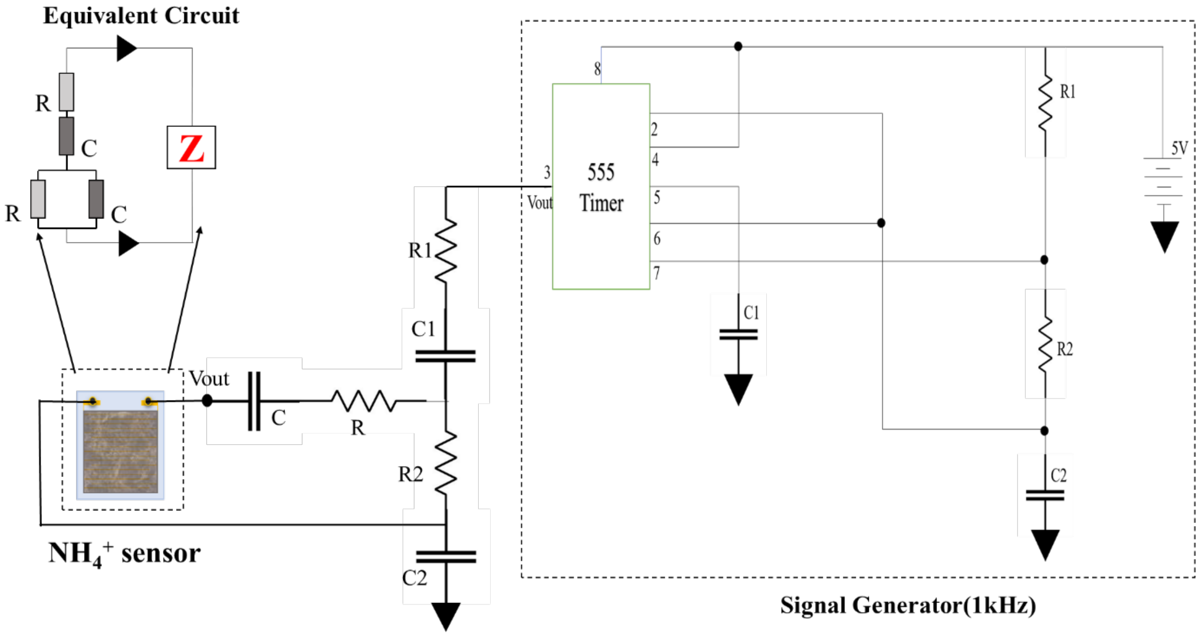

Fig. S1(b) Impedance characteristic measurement electronic circuit with 1 kHz frequency generator

EDS characterization:

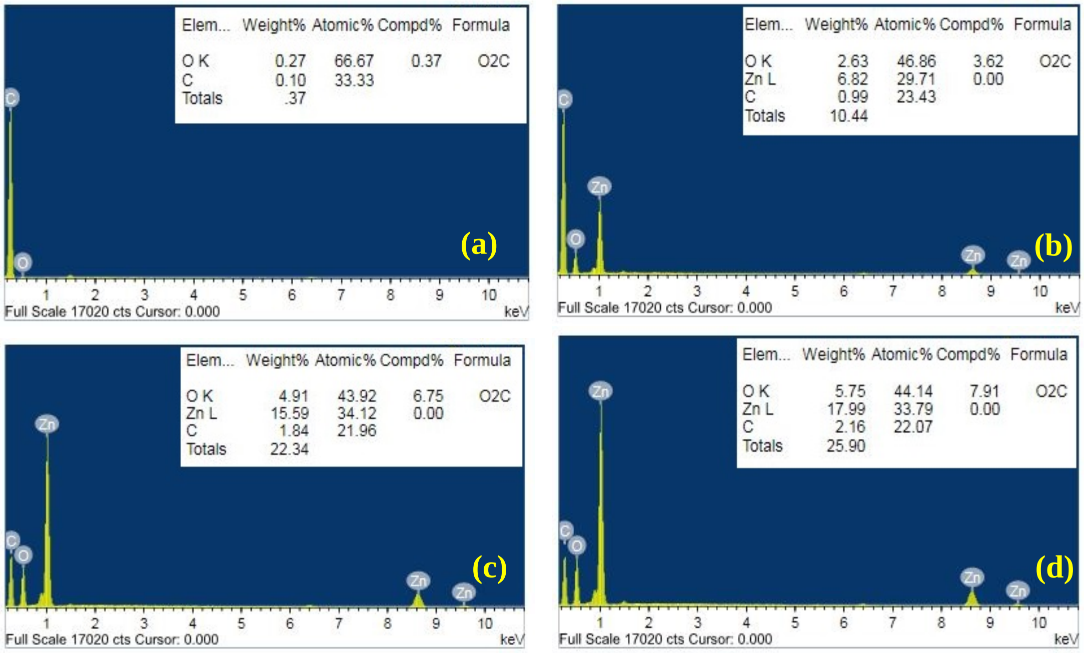

Fig. S2 EDS composition spectra for F-MWCNT (a), F-MWCNT/ ZnO-NFs composites with proportion of 1:1 (b), 1:3 (c) 1:5

Fig. S2 illustrates the EDS composition spectrum of the F-MWCNT (Fig. 4 a), F-MWCNT/ ZnO-NFs composites with proportion of 1:1 (Fig. 4 b), 1:3 (Fig. 4 c) and 1:5 (Fig. 4 d). The corresponding EDS spectrum confirms the presence of Carbon(C), Zinc (Zn) and Oxygen (O) elements in the F-MWCNT/ZnO-NFs nanocomposites and wright percentage of ZnO (6.82-17.99%) proportionally increasing with respect to the sample S1, S2 and S3. In addition, the result also indicates the higher distribution of the C and Zn in the scan area and no other impurity was detected in the chemical compositional studies.

XRD characterization:

No other peak related to impurities was detected in the spectrum, which further confirms the synthesized materials were of high purity and matches well with the standard crystallographic data's (ZnO: JCPDS 01-076-0704, C: JCPDS No. 00-026-1080). The strong diffraction peak at the angle 2θ of 25.58° can be indexed as the C (002) reflection of the hexagonal graphite structure and corresponding to the MWCNT, which confirms the formation of composites and completely reduced its peaks in the F-MWCNT/ZnO-NFs nanocomposites because of ZnO-NFs embedded on the entire surface of F-MWCNT. As a function of the ZnO concentration ratio, peak intensities of F-MWCNT and ZnO varies within nanocomposite samples (S1, S2 and S3). It is clear evident that on increasing the concentration of ZnO will decreases the peak intensity of the F-MWCNTs and ZnO peaks are becoming more intense. The peaks intensity of F-MWCNT/ZnO-NFs nanocomposites (S1, S2 and S3) decreased as compared with the untainted ZnO peaks. This indicates that the crystallinity of the materials decreases on composite formation.

Evaluation of electrochemical sensing performance of F-MWCNT/ZnO-NFs composites active layer:

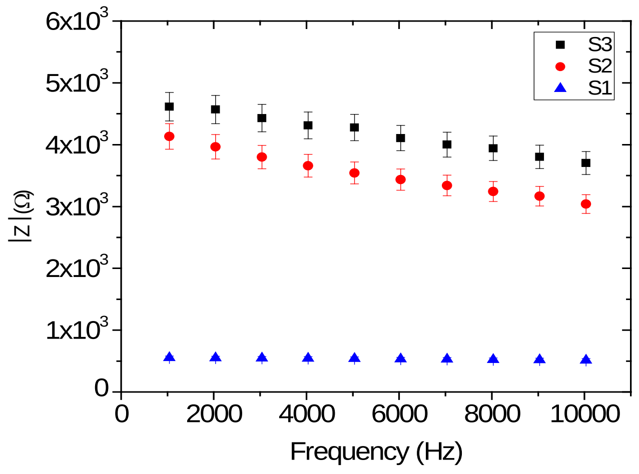

Fig. S3 Impedance magnitude versus frequency (kHz) response of the sensors (S1, S2 and S3)

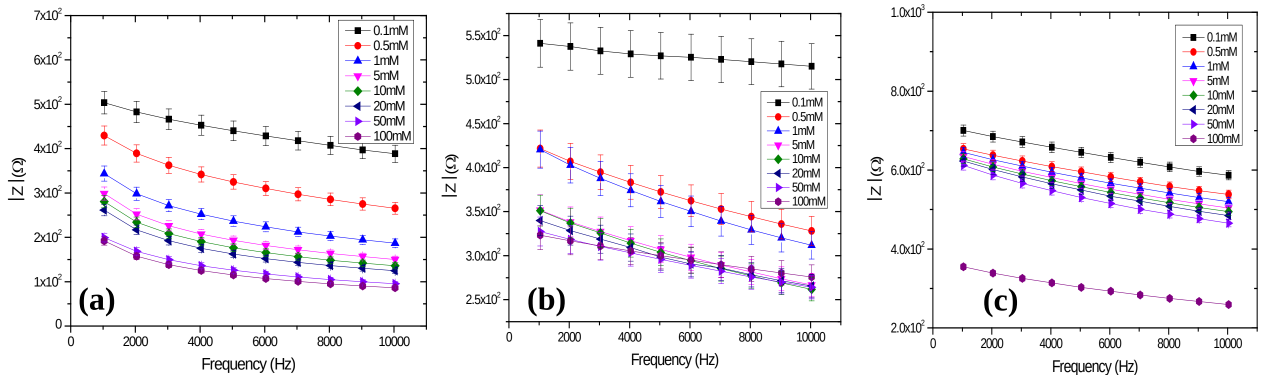

Fig. S4 Impedance magnitude (Ω) versus 0.1- 100 mM NH_4^+ion concentration response of the sensor S1 in the acid (a), neutral (b) and alkaline (c) sample environment conditions.

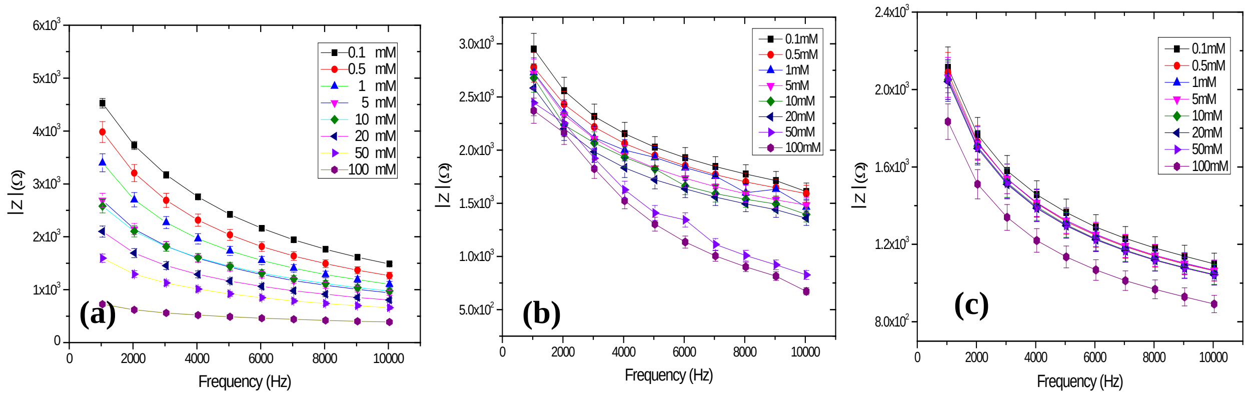

Fig. S5 Impedance magnitude (Ω) versus 0.1- 100 mM NH_4^+ion concentration response of the sensor S2 in the acid (a), neutral (b) and alkaline (c) sample environment conditions.

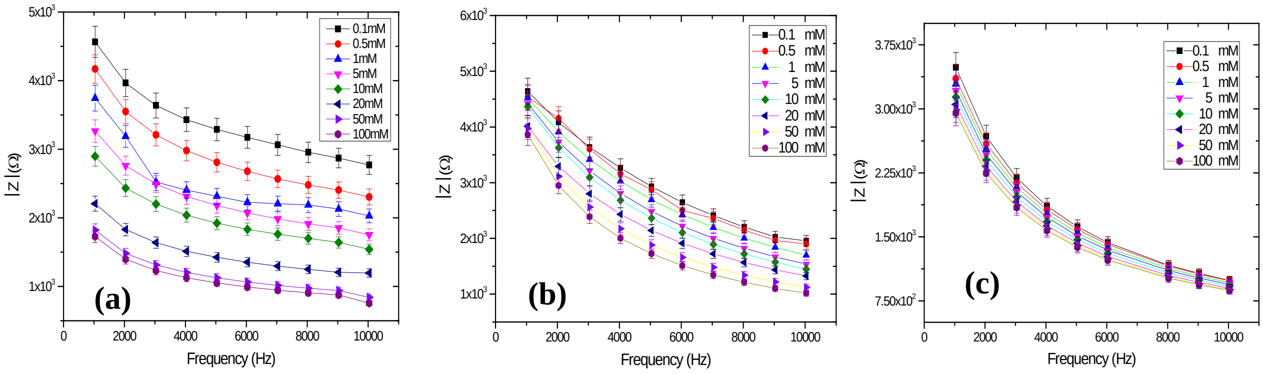

Fig. S6 Impedance magnitude (Ω) versus 0.1- 100 mM NH_4^+ion concentration response of the sensor S3 in the acid (a), neutral (b) and alkaline (c) sample environment conditions.

The fabricated sensors (S1, S2 and S3) sensitivity were estimated from the slope of the impedance magnitude versus NH_4^+ ion characteristics response from the calibration plots shown in Fig. S5 (a, b, and c), Fig. S6 (a, b, and c) and Fig. S7 (a, b, and c) respectively.

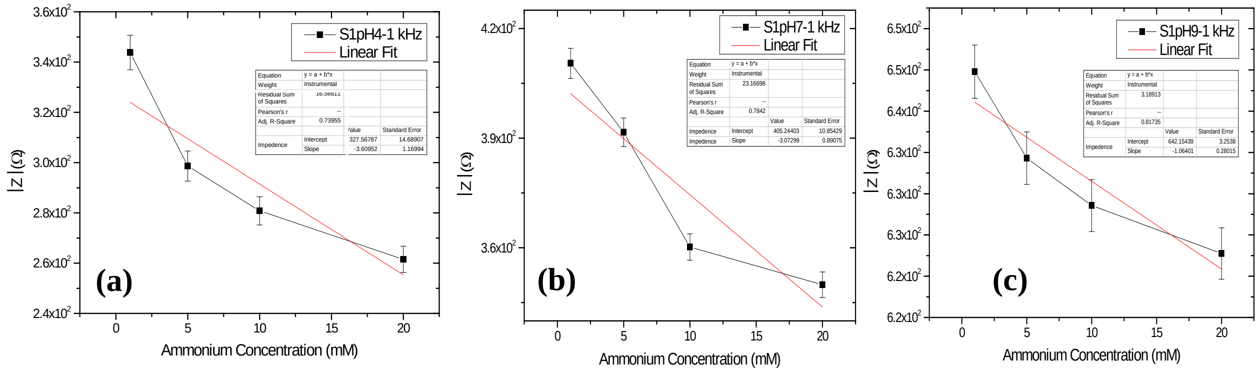

Fig. S7: Change in impedance magnitude versus NH_4^+ ion concentration (1-20 mM) response of the sensor S1 in the acid (a), neutral (b) and alkaline (c) sample conditions.

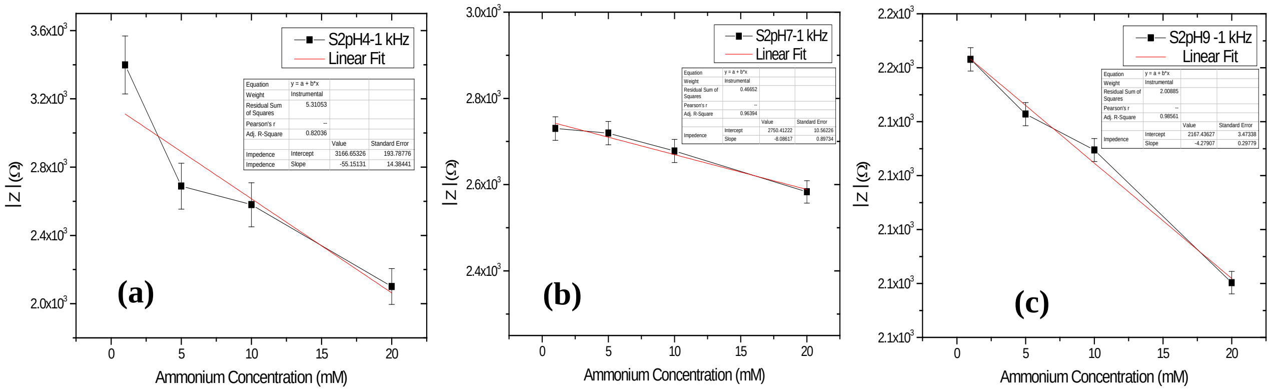

Fig. S8: Change in impedance magnitude versus NH_4^+ ion concentration (1-20 mM) response of the sensor in the acid (a), neutral (b) and alkaline (c) sample conditions.

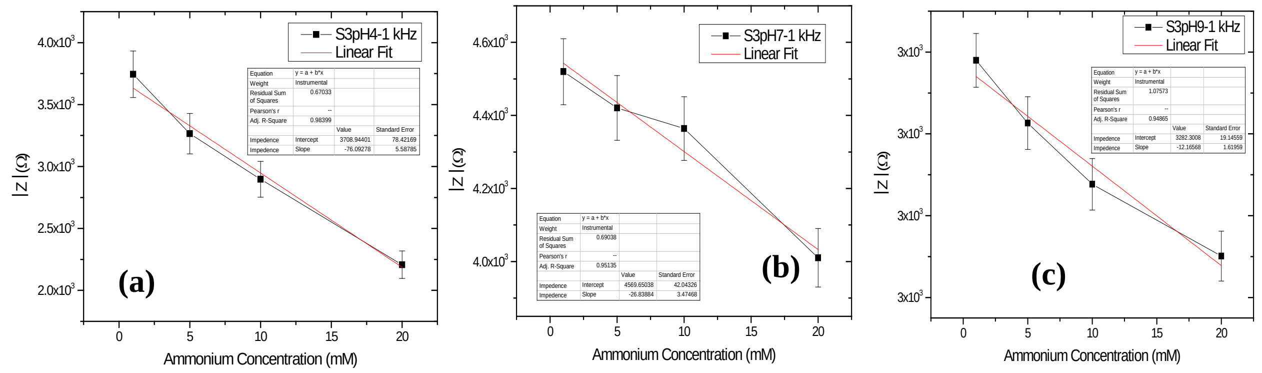

Fig. S9: Change in impedance magnitude versus NH_4^+ ion concentration (1-20 mM) response of the sensor S1 in the acid (a), neutral (b) and alkaline (c) sample conditions.

The correlation coefficient (R^2) and sensitivity of the fabricated device was calculated in the selective range and recorded in the Table S1

**NH_4^+ Sensor**	**NH_4^+ sample conditions**	**Correlation coefficient (R^2)**	**Sensitivity (mM/ Ω)**
S1	Acid (pH=4)	0.73955	3.60 ±0.18
	Neutral (pH=7)	0.78420	3.07 ±0.17
	Alkaline (pH=9)	0.81735	1.06 ±0.05
S2	Acid (pH=4)	0.82036	55.15 ±2.75
	Neutral (pH=7)	0.96394	8.08 ±0.40
	Alkaline (pH=9)	0.98567	4.27 ±0.21
S3	Acid (pH=4)	0.98399	76.09 ±3.80
	Neutral (pH=7)	0.95135	26.18 ±1.30
	Alkaline (pH=9)	0.94865	12.16 ±0.60

Table S1: Fabricated NH_4^+ sensor response for selective range about 1-20 mM with acid (a), neutral (b) and alkaline (c) sample conditions

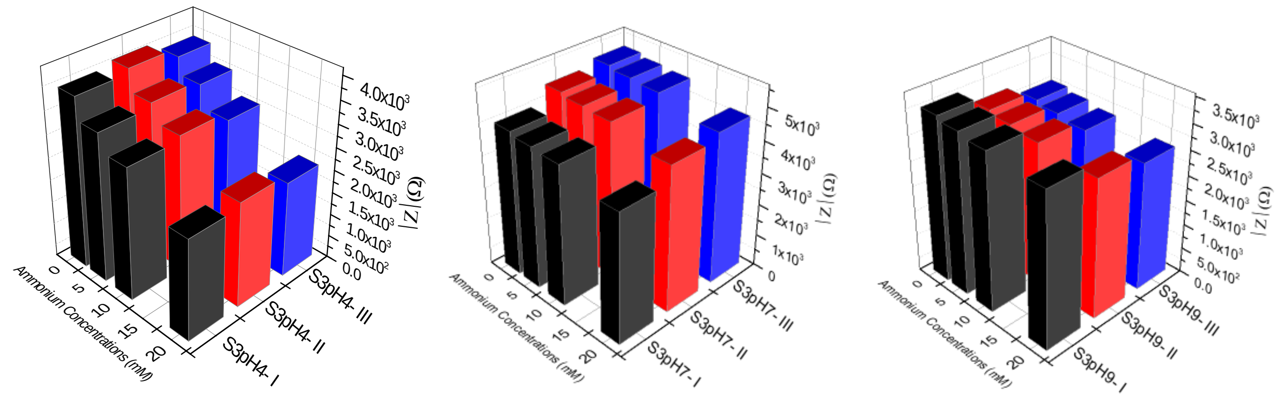

(a) **(b)** **(c)**

Fig. S10 Response of $|Z_{max}|$ versus NH_4^+ concentration (1 -20 mM) for three similar set of S3 devices in the acid (a), neutral (b) and alkaline (c) sample conditions

Machine learning based target predictions:

DS1

Scores

Model	AUC	CA	F1	Precision	Recall
kNN	0.8471345951629864	0.43157894736842106	0.4087938298047534	0.4341037548622688	0.43157894736842106
SVM	0.5322029442691903	0.3894736842105263	0.2845170618854829	0.3059298245614035	0.3894736842105263
Random Forest	0.9826498422712934	0.8315789473684211	0.8278736961081756	0.8458668730650154	0.8315789473684211
Neural Network	0.8537066246056781	0.45263157894736844	0.4207233770701263	0.42360681114551085	0.45263157894736844
Naive Bayes	0.7954784437434279	0.3368421052631579	0.32004668636247585	0.35930266843579534	0.3368421052631579
Logistic Regression	0.7706361724500526	0.3473684210526316	0.3079535424998155	0.3265350877192983	0.3473684210526316

Table S2: Model comparison at different sample sizes using various assessment metrics for Dataset 1

DS2

Scores

Model	AUC	CA	F1	Precision	Recall
kNN	0.8488433228180863	0.42105263157894735	0.40363649508386357	0.48262423671361615	0.42105263157894735
SVM	0.4733175604626709	0.3684210526315789	0.24852872001254378	0.29100478468899527	0.3684210526315789
Random Forest	0.9844900105152472	0.8631578947368421	0.8646132358760679	0.8789473684210526	0.8631578947368421
Neural Network	0.8388538380651945	0.45263157894736844	0.40500552079499447	0.44114844563760974	0.45263157894736844
Naive Bayes	0.7967928496319663	0.3684210526315789	0.37661130097972195	0.43958133971291863	0.3684210526315789
Logistic Regression	0.7237118822292324	0.3473684210526316	0.2537520367612929	0.20138852210351713	0.3473684210526316

Table S3: Model comparison at different sample sizes using various assessment metrics for Dataset 2

DS3

Scores

Model	AUC	CA	F1	Precision	Recall
kNN	0.8544952681388012	0.42105263157894735	0.3749863534136611	0.38696403766013276	0.42105263157894735
SVM	0.30796529968454256	0.3263157894736842	0.1798619499568594	0.221505376344086	0.3263157894736842
Random Forest	0.987973186119874	0.8315789473684211	0.826516134224947	0.8516655274550011	0.8315789473684211
Neural Network	0.8565983175604627	0.45263157894736844	0.3961270717720194	0.35678608285419433	0.45263157894736844
Naive Bayes	0.7954784437434281	0.3684210526315789	0.37661130097972195	0.43958133971291863	0.3684210526315789
Logistic Regression	0.6976866456361726	0.35789473684210527	0.2726101726464703	0.3151503759398496	0.35789473684210527

Table S4: Model comparison at different sample sizes using various assessment metrics for Dataset 3

		Predicted									
		**0.0**	**0.1**	**0.5**	**1.0**	**5.0**	**10.0**	**20.0**	**50.0**	**100.0**	**∑**
Actual	**0.0**	2	0	1	0	0	0	0	0	0	**3**
	**0.1**	0	9	0	0	0	0	0	0	0	**9**
	**0.5**	0	1	7	0	0	0	0	1	0	**9**
	**1.0**	0	0	1	8	0	0	0	0	0	**9**
	**5.0**	0	0	0	1	6	2	0	0	0	**9**
	**10.0**	0	0	0	0	0	9	0	0	0	**9**
	**20.0**	0	0	1	0	0	0	7	1	0	**9**
	**50.0**	0	0	1	0	0	0	0	28	0	**29**
	**100.0**	0	0	0	0	0	0	1	2	6	**9**
	**∑**	**2**	**10**	**11**	**9**	**6**	**11**	**8**	**32**	**6**	**95**

Table S5: Confusion matrix Random Forest (Showing number of instances)

		Predicted									
		**0.0**	**0.1**	**0.5**	**1.0**	**5.0**	**10.0**	**20.0**	**50.0**	**100.0**	**∑**
Actual	**0.0**	3	0	0	0	0	0	0	0	0	**3**
	**0.1**	0	5	2	1	0	0	0	1	0	**9**
	**0.5**	0	2	4	1	1	0	0	1	0	**9**
	**1.0**	0	2	1	2	1	0	0	2	1	**9**
	**5.0**	0	1	1	2	0	0	0	2	3	**9**
	**10.0**	0	1	1	0	2	0	0	2	3	**9**
	**20.0**	0	1	1	1	0	1	1	3	1	**9**
	**50.0**	0	0	1	1	0	0	0	23	4	**29**
	**100.0**	0	1	0	1	0	0	0	2	5	**9**
	**∑**	**3**	**13**	**11**	**9**	**4**	**1**	**1**	**36**	**17**	**95**

Table S6: Confusion matrix Neutral Network (Showing number of instances)

		Predicted									
		**0.0**	**0.1**	**0.5**	**1.0**	**5.0**	**10.0**	**20.0**	**50.0**	**100.0**	**∑**
Actual	**0.0**	1	2	0	0	0	0	0	0	0	**3**
	**0.1**	2	3	0	0	0	0	2	2	0	**9**
	**0.5**	1	2	0	0	0	0	2	2	2	**9**
	**1.0**	1	1	0	0	0	0	1	4	2	**9**
	**5.0**	0	2	0	0	0	0	1	4	2	**9**
	**10.0**	0	2	0	0	0	0	1	5	1	**9**
	**20.0**	0	1	0	0	0	0	2	4	2	**9**
	**50.0**	1	0	0	0	0	0	2	26	0	**29**
	**100.0**	1	0	0	0	0	0	1	6	1	**9**
	**∑**	**7**	**13**					**12**	**53**	**10**	**95**

Table S7: Confusion matrix Logistic Regression (Showing number of instances)

		Predicted									
		**0.0**	**0.1**	**0.5**	**1.0**	**5.0**	**10.0**	**20.0**	**50.0**	**100.0**	**∑**
Actual	**0.0**	3	0	0	0	0	0	0	0	0	**3**
	**0.1**	0	5	1	0	1	0	2	0	0	**9**
	**0.5**	0	4	3	0	1	0	0	1	0	**9**
	**1.0**	0	3	4	2	0	0	0	0	0	**9**
	**5.0**	0	3	2	0	3	0	0	1	0	**9**
	**10.0**	0	2	3	1	1	1	0	1	0	**9**
	**20.0**	0	2	2	1	2	0	0	2	0	**9**
	**50.0**	0	2	1	0	1	0	0	22	3	**29**
	**100.0**	0	2	1	1	2	0	0	2	1	**9**
	**∑**	**3**	**23**	**17**	**5**	**11**	**1**	**2**	**29**	**4**	**95**

Table S8: Confusion matrix kNN (Showing number of instances)

		Predicted									
		**0.0**	**0.1**	**0.5**	**1.0**	**5.0**	**10.0**	**20.0**	**50.0**	**100.0**	**∑**
Actual	**0.0**	3	0	0	0	0	0	0	0	0	**3**
	**0.1**	5	0	0	2	0	2	0	0	0	**9**
	**0.5**	4	0	0	3	0	1	1	0	0	**9**
	**1.0**	4	0	0	2	0	3	0	0	0	**9**
	**5.0**	2	0	0	3	0	4	0	0	0	**9**
	**10.0**	3	0	0	1	0	3	2	0	0	**9**
	**20.0**	2	0	1	1	0	1	4	0	0	**9**
	**50.0**	4	0	0	0	0	2	2	19	2	**29**
	**100.0**	3	0	0	0	0	0	2	0	4	**9**
	**∑**	**30**		**1**	**12**		**16**	**11**	**19**	**6**	**95**

Table S9: Confusion matrix Naïve Bayes (Showing number of instances)

		Predicted									
		**0.0**	**0.1**	**0.5**	**1.0**	**5.0**	**10.0**	**20.0**	**50.0**	**100.0**	**∑**
Actual	**0.0**	1	1	1	0	0	0	0	0	0	**3**
	**0.1**	0	4	0	0	0	0	0	5	0	**9**
	**0.5**	0	2	1	0	0	0	0	6	0	**9**
	**1.0**	0	1	1	0	0	0	0	7	0	**9**
	**5.0**	0	1	0	0	1	0	0	7	0	**9**
	**10.0**	0	1	0	0	0	0	0	8	0	**9**
	**20.0**	0	0	1	0	0	0	0	8	0	**9**
	**50.0**	0	0	1	0	0	0	0	28	0	**29**
	**100.0**	0	0	1	0	0	0	0	8	0	**9**
	**∑**	**1**	**10**	**6**		**1**			**77**		**95**

Table S10: Confusion matrix for SVM (Showing number of instances)

Code:

Input:
Super set-(Tri)={Tr1,Tr2….Tr5} Where Tr-Training Sets(i=1 to 5)
Super set(Tei)={Te1,Te2,….Te5} Where Te-Test Sets(i=1 to 5)
Machine Learning Algorithms :

NB ,RF,Knn,SVM,LR,NN(ML_i)

Where,

NB-Naive Bayes
RF-Random Forest
kNN-Knowledge nearest neighbor
SVM – Support vector machine
LR- Logistic Regression
NN-Neural Network

Output:

1, Best Approach Algorithm
2,Dominance in Feature Extraction
3,Predicted Values in best approach algorithms.

BEGIN

FETCH the datasets from source

```
    PREPROCESS the training & test datasets(DS_i) as Tr_i,Te_i
        CONVERT Categorical values(C_i) into Numerical values(Ni)
    FOR i=1 to 5 DO
        CHOOSE the target(T_i) variable in Tr_i
        CHOOSE the Original (Or_i) variable in Te_i
        FEED Features of (Tr_i) for FEATURE EXTRACTION(Fe_i)
        IDENTIFY the Fe_i values with Greater Probability to cover maximum  Fe_i values
        CHOOSE  the Higher Dominance of Fe_i from Tr_i
    END FOR
    FOR  j=1 to  6 DO
        FEED Fe_j into Machine learing Algorithms(ML_j)
        FIND the Predicted values(P_j) from Ml_j
    END FOR
    FOR i=1 to 5 DO
        IF Or_i== P_i THEN
            A_i=0
            A_i(Accuracy)=A_i + 1
        ELSE IF Or_i > P_i || Or_i< P_i THEN
            A_i(Accuracy)=A_i – 1
        END IF
    END FOR
    FOUND ACCURACY (A1,A2….A5)
    FOR i= 1 to 5 DO
        Gr_i=A_i
        IF A_i > Gr_i THEN
            Gr_i=A_i
        EVALUATE  greater (Gr_i) for the best Machine Learning algorithms
        END IF
    END FOR
END
```

INPUT/OUTPUT

We have Training set (Tr_i) and Test set (Te_i) as input for five datasets. Six Machine learning algorithms were tested to predict the target concentration in Test sets, and the most suitable algorithm is identified. Dominant predictors are extracted from all the features.

PREPROCESSING AND HIGHER DOMINANCE

Fetch Tr_i and Te_i from the source .Preprocess both datasets by converting categorical values (C_i) into numerical values(N_i). Target feature (T_i) from training sets (Tr_i) are selected. The original predicting feature (Or_i) from test sets (Te_i) are chosen. Predictors are extracted from the Training sets

(Tr_i) as Feature extraction (Fe_i). Identify the Fe_i values with the highest predominance (Fe_i) from Training sets (Tr_i).

ACCURACY FINDINGS

Feed the Higher dominance feature extracted values (Fe_j) values into the Machine learning algorithms (ML_j). Find the predicted values (P_j) of all algorithms (ML_j). Compare the accuracy of all algorithms based on Original value (Or_i) present in Test set and Predicted values (P_j). Evaluate and find Higher accuracy algorithms to choose the best algorithm in machine learning based the datasets collected from the source.
